# Emerging Roles of γ Aminobutyric Acid (GABA) Gated Channels in Plant Stress Tolerance

**DOI:** 10.3390/plants10102178

**Published:** 2021-10-14

**Authors:** Mona Kaspal, Madhuka H. Kanapaddalagamage, Sunita A. Ramesh

**Affiliations:** Transport Physiology and Signalling Group, Biological Sciences, College of Science and Engineering, Flinders University, Bedford Park, SA 5042, Australia; mona.kaspal@flinders.edu.au (M.K.); kana0060@flinders.edu.au (M.H.K.)

**Keywords:** γ-aminobutyric acid, aluminium-activated malate transporters, channels, receptors, signalling, GABA metabolism, stress response

## Abstract

The signaling role for γ-Aminobutyric acid (GABA) has been documented in animals for over seven decades. However, a signaling role for GABA in plants is just beginning to emerge with the discovery of putative GABA binding site/s and GABA regulation of anion channels. In this review, we explore the role of GABA in plant growth and development under abiotic stress, its interactions with other signaling molecules and the probability that there are other anion channels with important roles in stress tolerance that are gated by GABA.

## 1. Introduction

γ aminobutyric acid (GABA), a non-protein amino acid, was discovered nearly seven decades ago in potato tubers, followed by its discovery in mammalian brain extracts where it has been shown to be a major inhibitory neurotransmitter [1,2,3]. Activation of chloride conductance mediated through mammalian GABA_A_ receptors results in membrane hyperpolarisation in the central nervous systems, leading to dampening of neuronal firing [4,5,6,7,8]. The activity of GABA receptors in the brain and other organs in mammals has been extensively reviewed [6,7,8,9]. In plants, GABA is ubiquitous and has been documented to occur in many tissues and organs and its role was mainly defined as a metabolite [10,11,12,13]. However, in the last three decades, studies have shown that GABA concentrations range from micromolar (low) to millimolar (high) in different tissues, organs, and compartments, suggesting that it may be a signalling molecule, in addition to a metabolite in plants [11,14]. The role for GABA as a signalling molecule is further strengthened by the evidence that accumulation of GABA occurs in response to various abiotic and biotic stresses. Binding sites have been detected on cell membranes, GABA gated anion channels are present in plants, and their activity is regulated by agonists and antagonists of GABA receptors in animals [15,16,17,18,19].

The role of GABA in plant growth, development, and stress is well documented in numerous reviews, however the emerging evidence that ion channels implicated in plant stress have potential GABA binding sites and are regulated by it, merits re-examination of roles of GABA in signalling and stress tolerance [13,15,16,18,20,21,22,23]. In this review, we will explore various roles of GABA in plant growth and development briefly and focus on the effects of GABA gated ion channels on physiological processes in abiotic stress, explore other ion channels that may potentially be regulated by GABA, and investigate the implications of such regulation on stress tolerance *in planta*.

## 2. GABA Affects Physiological Processes

Synthesis of GABA occurs in the cytoplasm by decarboxylation of glutamate by glutamate decarboxylase (GAD) in the GABA shunt. The first GAD gene was isolated from *Petunia* and shown to have calmodulin binding domain (CaMBD) in the C terminus [24]. However, not all GAD genes have the CaMBD. In rice (*Oryza sativa*), *OsGAD1* can bind to CaMBD while *OsGAD2* does not bind to calmodulin [25]. In apple (*Malus x domestica* Borkh.), recombinant *MdGAD1* and *2* have been shown to bind to calmodulin while *MdGAD3* does not bind to it [26]. In tea (*Camellia sinensis*) leaves, recombinant *CsGAD1* and *2* expressed in *E. coli* convert glutamate to GABA while *CsGAD3* showed no activity [27]. *CsGAD1* has calmodulin regulated autoinhibitory domain while *CsGAD2* and *3* have calmodulin independent autoinhibitory domain. Further, the expression of *CsGAD2* was upregulated by mechanical damage caused by picking tea leaves [27].

It has been well documented that GABA can improve or inhibit plant growth and development [28]. In duckweed (*Lemna minor* L.), the addition of 5 mM GABA to the nutrient solution caused a two-fold increase in plant growth than the nutrient solution alone [18,29]. Gradients of GABA in the stigma and style are important for proper growth of the pollen tube to reach the ovary, impaired gradients affect fertilisation [30]. Studies in *Arabidopsis* and *Brassica napus* suggest a possible role for GABA in regulating nitrate uptake and affect nodule formation in *Medicago* [31,32,33,34,35,36]. GABA is involved in regulating leaf senescence and studies suggest that fluctuations in endogenous GABA levels may be regulated or regulate the plant circadian clock [37,38,39]. Disruption of GABA transaminase (GABA-T) severely impaired cell elongation in *Arabidopsis* pollen tubes, primary roots, and hypocotyls [30,40,41]. Similarly, overexpression of a *Petunia GAD* gene (C-terminal calmodulin binding domain removed) in tobacco resulted in increased concentrations of GABA over the wild type and caused slow growth, increased branching and shorter cortical parenchyma [28]. Higher levels of reactive oxygen species (ROS) were observed in *Arabidopsis* when the succinic semialdehyde dehydrogenase (*SSADH*) gene was disrupted [12].

Multiple abiotic stresses increase endogenous GABA concentrations in the tissues and, as exogenous application of GABA mimics the effects of stress on growth and development, it provides an easy method to investigate its role in plants [42]. The speed and rate of stress induced GABA accumulation varies from seconds to days and from 10 to 1000-fold in different plant species - reviewed in these references [43,44,45,46,47,48,49,50,51,52,53,54,55]. Among the abiotic stresses, salinity and drought have been widely studied in numerous plant species including *Arabidopsis*, barley, rice, *Medicago*, tobacco, soybean and *Populus* [39,40,44,47,49,56,57]. In maize seedlings exposed to salt (150 mM NaCl), application of GABA (0.5 mM) resulted in improved growth, alleviated damage to cell membranes, increased soluble sugars and proline accumulation, and reduced water loss [58]. Exposure to 150 mM NaCl led to GABA accumulation and inhibition of primary root growth (17%) in *Arabidopsis* GABA-T mutant [59] while in *Arabidopsis gad1/gad2* mutants decrease in GABA synthesis resulted in increased stomatal conductance rendering the plants more sensitive to drought [55]. A triple mutant *gad1/gad2 X pop2-5* was observed to have increased endogenous GABA content with stomatal conductance restored to wild type levels and was less sensitive to drought [55]. Under hypoxia, exogenous application of GABA in *Prunus* genotype, sensitive to waterlogging, led to increased stomatal conductance, lower ROS production, and less leaf lesions when compared to the tolerant genotype [60]. In sunflower, GABA (2 mg/L) treatment increased chlorophyll and sugar content, resulting in the upregulation of antioxidant enzymes during heat and drought stress [61]. The application of GABA (50 mg/L) to wheat seedlings limited ROS production, increased accumulation of soluble protein and maintained amino acid homeostasis, while the application of GABA (1 mM) to heat stressed mung beans improved reproductive function [62,63].

Cold stress can severely effect plant growth and yield and several studies have shown that accumulation of GABA, proline, and polyamines contribute to chilling tolerance [64]. Cold sensitive tomato plants treated with GABA (50 mM) showed improved plant growth by regulating antioxidant levels and cell expansion [65]. Treatment of fruit with GABA before cold storage has been shown to improve chilling induced injuries and improve shelf-life [66,67,68,69,70]. All these studies indicate that GABA has an important role to play not only in normal growth and development but also during the diverse abiotic stresses that plants encounter, stresses that are set to increase in frequency due to adverse growing conditions resulting from global climate change.

## 3. GABA Regulation of Gene Expression

Studies have documented GABA regulation of gene expression and possible interactions with other signalling and stress response pathways [71,72]. In *Arabidopsis*, GABA downregulates the expression of family of *14-3-3* genes [71]. This family of genes encode ubiquitous regulatory proteins that regulate target proteins in a phosphorylation dependent manner and have important roles in regulating carbon and nitrogen metabolism in plants. The *14-3-3* proteins interact with calcium dependent protein kinases, sucrose non-fermenting -1- related protein kinase 1, other protein kinases, and nitrate reductase [71]. GABA represses the transcription of most of the family of *14-3-3* genes in the presence of high external calcium and interestingly this response is dependent on intact ethylene and abscisic acid signalling pathways suggesting a possible cross talk [71].

In *Brassica napus*, exogenous GABA (100 μM) application to the roots upregulated the mRNA expression of nitrate transporter *BnNrt2* but did not have a significant effect on nitrate influx [31]. In rice seedlings treated with either 50 or 100 μg/mL of GABA, accumulation of arsenic was decreased and at lower concentration induced the expression of *Lsi-1* transporter in the roots [73]. Interestingly the addition of GABA (50 μg/mL) along with arsenic downregulated the expression *Lsi-1* gene in the roots, whereas the expression of *Lsi-2* was downregulated by addition of 100 μg/mL GABA along with arsenic. The expression of various antioxidant enzymes such as catalase, POD, SOD, and MDHAR were higher in GABA treated plants [73].

Under salinity, the leaves of *Agrostis stolonifera* (creeping bentgrass) had lower expression of inorganic pyrophosphatase (*AsPPa2*), sodium hydrogen exchanger (*AsNHX1*, *AsNHX4*), salt overly sensitive (*AsSOS1*), and sodium transporter (*AsHKT4*) genes while roots showed increased expression of *AsNHX1, AsNHX2*, *AsNHX4*, *AsNHX6*, *AsNHX8*, and *AsSOS20* in GABA treated plants compared to the control plants [74]. GABA-treated plants also exhibited significantly higher *AsSOS1* expression. All these genes are involved in Na^+^ transport, efflux, and compartmentation.

**Figure 1:**
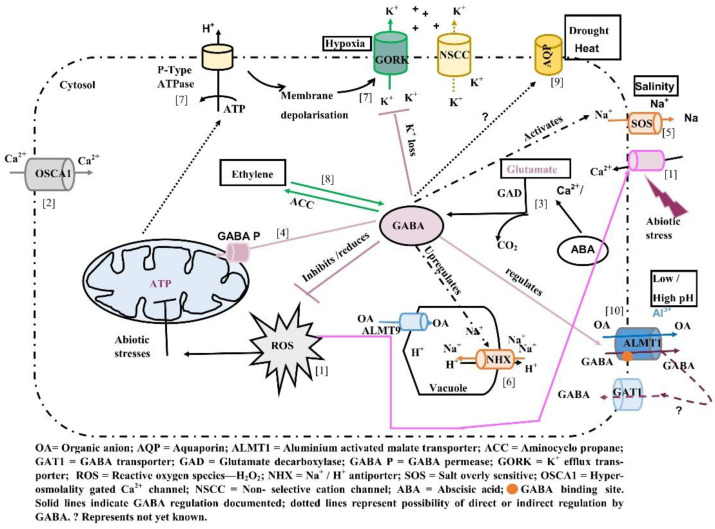
Summary of known ion channels and transporters whose activity is regulated by GABA and potential candidates (ion channels) whose activity may be regulated by GABA under various abiotic stresses (for description, see text).

Although it is known that salt stress limits water uptake mediated by aquaporins (*AQP*s), and GABA concentrations increase under salinity, it is unclear whether GABA is involved in the regulation of activity of aquaporins (Figure 1). Exogenous GABA application in creeping bentgrass alleviated drought-induced decline in relative water content and water use efficiency and increased organic acids in leaves [75]. GABA application significantly downregulated transcript levels of aquaporins *TIP1-1*, *TIP1-2*, *TIP2-1*, *PIP1-1*, and *NIP1-4* under salt stress [74].

## 4. GABA Is Involved in Cross Talk with Other Stress Signalling Pathways

Phytohormones such as auxins, cytokinins, abscisic acid, and ethylene interact with GABA and may have a role in stress tolerance [72]. Both indole acetic acid (IAA, 10 μM) and abscisic acid (ABA, 100 μM) have been shown to induce the transcription of *Arabidopsis* Aluminium Activated Malate Transporter *AtALMT1,* which is essential for aluminium (Al^3+^) tolerance [76]. GABA (100 μM) can regulate the malate stimulated currents in *AtALMT1* expressed in *Xenopus* oocytes [16].

Transcriptomic analysis of *Arabidopsis* seedlings grown on GABA (10 μM) revealed the downregulation of a number of genes encoding lipid transfer proteins, peroxidases, and cell wall proteins such as arabinogalactans [77]. Interestingly, application of cytokinin (6-benzyl adenine) to *Arabidopsis* seedlings downregulated the same set of genes affected by GABA treatment. Tobacco plants transformed with iso-pentyl transferase (*ipt*) driven by senescence activated promoter (SAG) showed increased cytokinin content and accumulation of proline, methionine, and GABA when exposed to increasing levels of zinc in the soil [78].

Abscisic acid (ABA) is a critical hormone involved in plant growth and development and regulates plant responses to stress [79]. The ABA levels change in response to various abiotic stresses (e.g., drought, heat, salinity) and play crucial roles in stomatal closure mediated by signalling cascades involving pyrabacin resistance 1(*PYL*)/ *PYR1*-like regulatory components of ABA receptors (RCAR), type 2C protein phosphatases (PP2Cs), sucrose non-fermenting 1-related subfamily 2 (*SnRK2*s) and protein kinase open stomata 1 (*OST1*) [80]. *Arabidopsis* plants deficient in GABA synthesis showed deformed stomata and impaired stomatal closure [55]. Recently, it has been reported that guard cell GABA production is essential for reducing stomatal opening and transpirational water loss by negative regulation of tonoplast localised At *ALMT9* in *Arabidopsis* [81]. GABA (2 mM) was observed to inhibit ABA (2.5 μM) induced stomatal closure at low concentrations and in mutant plants impaired in GABA synthesis (*gad2-1*) and *ALMT9* (*almt9*), ABA could induce stomatal closure to wild type levels [81]. Taken together, these findings suggest that there is a cross talk between GABA regulated *ALMT9* and ABA in mediating stomatal closure under drought stress (Figure 1).

Levels of ethylene, another phytohormone are regulated in response to stress [82,83]. Application of increasing concentrations of GABA (0–300 mM) promoted ethylene production (14 fold) in sunflower seedlings by upregulation of the expression of 1-aminocyclopropane-1-carboxylic acid (ACC synthase) [84]. Many stresses such as wounding, hypoxia, cold and heat under which plants evolve more ethylene, also result in GABA accumulation. Studies suggest that during wounding or mechanical damage, GABA accumulation occurs within 30 s and precedes ethylene production which occurs after 30 min [84]. Malate efflux from tobacco BY2 cells expressing wheat *ALMT1* was inhibited when cells exposed to Al^3+^ were treated with ethylene donor Ethrel [85]. Further, ethylene evolution and malate efflux from root tips increased in Al^3+^ tolerant near isogenic of wheat ET8 but malate efflux was inhibited on exposure to ethylene precursor ACC [85].

Many of the abiotic stresses under which the levels of phytohormones change also result in changes in GABA levels suggesting that there may be interactions between multiple players and pathways that mediate plant stress tolerance.

## 5. GABA Gated Channels

Many studies have well documented the role of GABA as a primary metabolite, but its signalling role is an emerging area of research in plants [11,13,15,16,30]. For a molecule to be classified as a signal, it needs to be present in very low concentrations, bind to a receptor, and elicit a specific cellular response. The fact that GABA occurs in high concentrations and is present in almost every part of the plant would argue against it being a signal [11]. Molecules that lead to changes in membrane potential in response to a triggering event are key cellular signals and, as GABA modifies membrane potential and is essential for plant growth, it has a potential signalling role *in planta* [16]. Early evidence for a signalling role for GABA was observed in bacterial quorum sensing [86]. Numerous other studies reported involvement of GABA in regulation of stomatal apertures under drought, plant-insect communication, plant-bacterial interactions, regulation of ROS levels and modulation of growth and development by exogenous application leading to the speculation that it has a signalling role in plants [14,22]. The discovery of family of plant glutamate receptors (*GLR*s) led to the assumption that GABA might function as a signal via *GLR*s [87,88]. However, no *GLR* to date has been shown to interact with GABA.

Evidence for GABA regulation of transporters involved in tolerance to aluminium toxicity, salinity, and hypoxia is just beginning to emerge (Figure 1). (1) Abiotic stresses such as salinity, cold, heat, and drought can lead to increase in cytosolic Ca^2+^ levels via calcium influx mediated by Ca^2+^ channels and the production of reactive oxygen species (ROS). Increasing cytosolic Ca^2+^ levels activate ROS generating enzymes leading to ROS production. However, generation of ROS during stress may also activate Ca^2+^ uptake channels leading to increase in cytosolic calcium levels [23,89,90]. (2) *OSCA1* has been identified as an osmosensor localised to the plasma membrane involved in increases in internal calcium in response to osmotic stress [91]. (3) Increases in internal Ca^2+^ lead to Ca^2+^ binding to calmodulin binding site (CaMBD) on glutamate decarboxylase (*GAD*) leading to conversion of glutamate to GABA. Under stress, key enzymes of the tricarboxylic acid cycle (TCA) are inhibited. (4) Increased GABA produced in the cytosol is transported to the mitochondria via GABA permease (GABA P) and provides anaplerotic succinate to maintain energy production via ATP synthesis [12,92]. (5,6) GABA may be involved in modulating the activity of SOS genes involved in mediating Na^+^ efflux and *NHX* antiporters in sequestering excess Na^+^ into the vacuole [74]. (7) Accumulation of GABA under stress reduced Na^+^ uptake, ROS concentrations, induced the activation of H^+^ ATPase and decreased K^+^ loss [23,75,93,94,95]. (8) Exogenous GABA application influences ethylene biosynthesis via changes in transcript abundance of ACC synthase and ACO (ACC oxidase), key enzymes in the biosynthetic pathway [84,94]. (9) The expression of some aquaporin genes has been observed to change in response to changing GABA levels in the cells under stress, however whether these genes are regulated by GABA requires further investigation [74]. (10) Recently, GABA has been shown to regulate the activity of *ALMT* family of proteins influencing responses to stresses such as drought, acid, and alkaline soils [16,84,96]. Thus, accumulation of GABA under stress may influence the expression and activity of key enzymes and ion channels and play a role in mediating plant stress tolerance.

The recent discovery of a putative GABA binding site (12 amino acids) on a family of plant anion channels–ALMTs with homology to the binding site on the mammalian GABA_A_ receptor has provided the first experimental evidence towards establishing GABA as a signalling molecule [16]. This discovery has been referred to as the discovery of ‘GABA receptor’ in plants [97]. The ALMT proteins mainly encode voltage gated anion channels but also include rapid or quick acting anion channels [96,98,99,100]. The *ALMT* family is multigenic and members of this family are involved in processes such as aluminium tolerance (*TaALMT1*), mineral nutrition and ion homeostasis (*ZmALMT1*), vacuolar homeostasis (*AtALMT9*), stomatal movement (*AtALMT12*), and solubilisation of soil nutrients such as phosphate (*ZmALMT2*) [101,102,103,104,105].

Opening of *ALMT* anion channels leads to release of anions (malate) and depolarisation of the membrane [106]. The *ALMT*s can also be transactivated by anions such as sulphate on the efflux side of the channel protein [16,98]. Although both *ALMT*s and GABA_A_ receptors encode anion channels, they share little similarity in the full-length protein sequences. The putative GABA binding site on the *ALMT*s is a 12 amino acid stretch occurring at the end of sixth transmembrane domain and towards the C terminus of the proteins [14,16]. GABA regulated anion (malate) efflux mediated by the ALMTs is sensitive to low micromolar concentrations of GABA [16]. Further, the activation of wheat *ALMT1* (*TaALMT1*) leads to a negative correlation between anion (malate) efflux and endogenous GABA concentrations in heterologous expression systems *in planta*. Aromatic amino acids phenylalanine (F) and tyrosine (Y) in the putative GABA binding site are important for GABA regulation and mutation of these amino acids to cysteine effects GABA affinity and increases the EC_50_ from 3.4 μM to 1.8 mM in the wheat *ALMT1* and from 6.0 μM to 380 μM in the *Vitis ALMT9* [14,16].

Interestingly the correlation between anion efflux and GABA concentrations is uncoupled in *TaALMT1* when phenylalanine is mutated to cysteine (F213C) [16,107]. Decrease in endogenous GABA concentrations is a result of GABA efflux mediated by *TaALMT1* and *ALMT*s can both transport and efflux GABA [107]. Patch clamping of *TaALMT1* in *Xenopus* oocytes suggests that GABA inhibits malate currents from the cyto-plasmic side [108]. The ability of *ALMT*s to transport GABA and be regulated by it involves a fine balance between the need for anion efflux under adverse conditions such as aluminium toxicity and the necessity to regulate such flux to prevent carbon and nitrogen loss. It is unknown whether the transport of anions such as malate and GABA via *ALMT*s occur due to a switch between anion conducting or GABA conducting states in the same pore or due to conformational changes occurring due to the binding of GABA to the protein.

Recently, it has been shown that GABA regulates the tonoplast localised *Arabidopsis AtALMT9* and plasma membrane localised *AtALMT12* in the guard cells and modulates the opening and closing of stomata under drought [81]. This opens avenues for further research into cross talk between signalling pathways that are involved in regulation of stomatal apertures in response to various abiotic stresses and whether there are other *ALMT*s that may be involved in mediating such responses. The discovery of GABA-gated channels provides compelling evidence that GABA is a plant signalling molecule and these putative plant ‘GABA receptors’ are involved in translating changes in metabolic status into physiological responses during stress [109].

It is well known that depolarisation activated outward rectifying potassium (K^+^) efflux channels (*GORK*s) from *Arabidopsis* have an important role in stress induced K^+^ efflux from plants cells [110]. These voltage gated channels are expressed in guard cells and are involved in stomatal closure [111,112]. Recently it was shown that both *GORK* channels from plants and the mammalian outward rectifying K^+^ efflux channels (ORKs) have a putative GABA binding motif that is similar to that identified in ALMT proteins [113]. In *Arabidopsis* root epidermis, GABA induced K^+^ efflux was markedly different between wild type (Col0) and *gork*1-1 mutant impaired in K^+^ efflux when measured via the Microelectrode Ion Flux Estimation (MIFE) technique [113]. The authors suggest that GABA regulation of K^+^ efflux via *GORK*s is important for stress tolerance. Further, greater salinity tolerance of *Arabidopsis* mutant *pop2-5* (GABA-transaminase knock out) that is impaired in GABA catabolism has been in part attributed to higher concentrations of GABA, stress-induced activation of H^+^ ATPase leading to efficient maintenance of transmembrane electrical potential, and reduced stress induced K^+^ leak mediated by *GORK*s from the roots [93].

Hypoxia induced accumulation of GABA is large and has been documented in number of plant species [43,63]. In a study with *Arabidopsis* wild type (Col0) and mutants impaired in GABA synthesis (*gad1/2*), plants with less GABA accumulation were observed to be less tolerant to hypoxia, had lower fresh weight and chlorophyll content compared to wild type plants [23]. Hypoxia induces ROS (H_2_O_2_) accumulation leading to loss of cell viability and, interestingly, in this study it has been shown that higher concentrations of GABA in the *pop2-5* mutants resulted in lower levels of H_2_O_2_ when compared to wild type or *gad1/2* plants. Waterlogging leads to reduced oxygen availability affecting H^+^-ATPase pump activity resulting in membrane depolarisation and K^+^ loss via *GORK* channels [23]. Increased GABA shunt activity leading to accumulation of GABA under hypoxia may restore membrane potential by regulation of H^+^-ATPase activity as synthesis of GABA consumes a proton (H^+^) and raises pH [114]. Restoration of negative membrane potential is essential to prevent stress induced K^+^ efflux and, thus, increased GABA levels may regulate K^+^ loss via the *GORK* channels [23,115].

Transporters involved in sodium (Na^+^) transport and efflux are important for maintaining ion homeostasis under salt stress. Transcripts levels of numerous genes involved in Na^+^ transport and compartmentalisation were observed to change in GABA treated *A. stolonifera* under salinity, however, this study did not provide any evidence for GABA regulation of genes encoding these transporters or channels [74]. It would be interesting to express some of these transporters/channels in heterologous expression systems and investigate if they are regulated by exogenous GABA and whether their regulation by GABA contributes to salinity tolerance in plants. GABA accumulation occurs under both drought and salinity, and it is known that salt stress limits water uptake. However, there is no evidence yet whether GABA is involved in the regulation of aquaporins that mediate water uptake.

## 6. Conclusions

Emerging evidence indicates that GABA may have a dual role, namely that of a metabolite and a signal under stress. *ALMT*s are the first plant proteins that have been shown to have a putative GABA binding site. GABA regulates malate efflux mediated by *ALMT*s in response to acidic and alkaline stresses and modulates stomatal opening to improve water use efficiency under drought stress. As GABA concentrations increase when plants are exposed to diverse abiotic stresses, it is highly likely that other transport proteins or channels (e.g., aquaporins) involved in abiotic stresses (e.g., salinity, drought) may be regulated by GABA either directly or indirectly (Figure 1). Not much is known about how GABA binds and interacts with proteins (e.g., whether the generated signal is transient or sustained) or the molecular basis of GABA regulation of transporters and ion channels that help plants tolerate and thrive under adverse conditions. No functional characterisation of recently identified GABA motif in *GORK* channels has been carried out yet. Mutational studies could pinpoint residues essential for GABA binding and affinity of GABA for binding to these residues. These knowledge gaps need to be bridged to enhance our understanding of the structural and molecular basis of stress-induced GABA regulation of various proteins to improve plant productivity for future food security.
